# Mindfulness-Based Attention Training in the Navy: A Feasibility Study

**DOI:** 10.1177/00332941231154442

**Published:** 2023-02-01

**Authors:** Anja Roemer, Anna Sutton, Carsten Grimm, Stafford Kimber, Oleg N. Medvedev

**Affiliations:** 6420 Massey University, School of Psychology, Palmerston North, New Zealand; 3717 University of Waikato, School of Psychology, Hamilton, New Zealand; 8468 New Zealand Defence Force, Wellington, New Zealand; 3717 University of Waikato, School of Psychology, Hamilton, New Zealand

**Keywords:** mindfulness-based intervention, sustained attention, well-being, military training

## Abstract

Mind wandering is common during daily activities and is even more prevalent under stressful conditions, which could lead to lapses in attention and poor performance. Newly recruited military personnel who undergo demanding training often experience high levels of stress. It is therefore imperative to find ways to foster mental health and avoid performance deterioration related to mind wandering in times of intense military training. This feasibility study investigated the effectiveness of an established low-dose mindfulness-based intervention (MBI), called Mindfulness-based Attention Training (MBAT), on mind wandering, attentional performance, and well-being, delivered by a facilitator who was taught how to deliver MBAT. A sample of newly recruited Royal New Zealand Navy (RNZN) Junior Officers (*n* = 17) undergoing demanding training participated in the 8-week long MBI with one weekly contact session. Measures of well-being and the Sustained Attention to Response Task (SART) were completed 4 weeks prior to the MBAT, at the start of the MBAT, at the end of the MBAT and 4 weeks after completion of the MBAT. Results suggest that MBAT might protect from performance decline during intense training and enhance levels of well-being at follow-up. These findings highlight the valuable role of mindfulness as a component in military training.

## Introduction

When engaging in daily activities, such as driving, reading or executing tasks at work, our attention to the task at hand tends to drift away and shifts to thoughts that are not related to what we are doing in the present moment. This phenomenon is called mind wandering, defined as stimulus-independent and task-unrelated thoughts (Stawarczyk et al., 2012). Forty-two percent of employees report that they experience problems focusing on tasks at work (Hougaard, 2020) and findings from an experiential study even suggest that 30% of people experience mind wandering during every reported activity (Killingsworth & Gilbert, 2010). Attentional lapses associated with mind wandering can have negative, even disastrous consequences. This can be the case in aviation (Jones & Endsley, 1996), health care (Fore & Sculli, 2013), or the military (Jha et al., 2015) when individuals are required to suddenly adjust behaviour but do not manage to do it, for instance if pilots fail to react to a technical emergency, nurses do not correctly apply medication to patients in a deteriorating condition, or deployed soldiers accidentally harm civilians while in a conflict zone. Considering that mind wandering is more often reported when experiencing stress (Crosswell et al., 2020), it is important to address mind wandering in highly demanding and stressful professions to prevent negative performance outcomes. Jha et al. (2015) for example found that military personnel going through demanding predeployment training showed more attentional performance lapses over time. Subjective, self-reported mind wandering statistics are high (Gilbert, 2010), but it is likely that they underestimate the actual frequency of mind wandering in everyday life. Because mind wandering reflects disengagement of attention from perception as well as a lack of meta-awareness, which is the individual’s knowledge of current thoughts (Schooler et al., 2011), it is likely that individuals cannot accurately capture and report their own mind wandering and thus, more objective measures of mind-wandering are needed. Objective measures of the attentional lapses that occur during mind-wandering, and the impact of these on performance, can give a clear picture of how mind-wandering affects performance. Computer-based programmes such as the Sustained Attention to Response Task (SART; Robertson et al., 1997) can provide these objective measures by evaluating the person’s ability to sustain attention throughout a task (Jha et al., 2015; Zanesco et al., 2019). Sustained attention is “the ability to self-sustain mindful, conscious processing of stimuli whose repetitive, non-arousing qualities would otherwise lead to habituation and distraction to other stimuli” (Robertson et al., 1997, p.747). If attention is not sustained, individuals fail to respond to stimuli change, which can be considered an attentional lapse (Jha et al., 2015) and greater variation in response reaction times when responding to changing stimuli is a valid indicator of mind wandering (Seli et al., 2013). Sustained attention involves being mindful (Robertson et al., 1997) that is, being aware of present moment experiences (Kabat-Zinn, 2003) and indeed mindfulness is negatively correlated with both subjective self-report and objective measures of mind wandering (Mrazek et al., 2012). Mindfulness-based interventions (MBIs), which train participants in mindfulness, may therefore be a way to reduce mind wandering. Many MBIs applied in workplace settings are based on the principles of mindfulness-based stress reduction (MBSR) programmes (Kabat-Zinn, 1990), which are rather time-consuming, involving a weekly 2.5-hour contact session, 45 minutes of home practice on 6 days a week and often a full-day retreat (Jamieson & Tuckey, 2017). It might be difficult to implement this protocol in demanding and busy jobs, therefore shorter, similarly effective MBI protocols are desirable. Several studies have found that abbreviated, low-dose protocols show positive results in terms of attentional performance. For example, an 8-hour MBI distributed over 8 weeks showed positive effects on attentional performance in military cohorts undergoing demanding military training. Trainees who received the MBI did not show the decline in attentional performance over time that trainees showed who received no MBI or didactic-focused mindfulness training (Jha et al., 2015). These results indicate that MBIs may protect participants in highly demanding working conditions from performance deterioration over time. Similarly, a 4-week MBI with weekly 2-hour sessions can enhance attentional performance on the SART compared to a control group (Zanesco et al., 2019). The effect of mindfulness training on attentional performance and mind wandering has mostly been conducted with groups exposed to demanding work situations who are already fairly skilled and trained, such as elite soldiers (Zanesco et al., 2019) or athletes (Rooks et al., 2017). However, individuals who are new to a profession and receive intensive training to acquire the needed skills also experience high levels of stress, especially new military recruits who enter a completely new environment that is disconnected from their family and other established support networks, which can negatively affect their well-being (Cigrang et al., 2000). In this critical stage of training, it is important to support well-being in addition to performance to avoid early dropouts. Mindfulness training was found to have positive effects on health and stress of new army recruits (Guo et al., 2019) and unemployed youth attending an intensive military training camp (Roemer et al., 2021), indicating that MBIs can have beneficial mental health effects. Considering that military trainees often experience demanding and stressful environments, effective interventions for enhancing well-being, reducing mind wandering, and protecting attentional performance are needed. This study aimed to examine the effects of a low-dose MBI, developed by Jha et al. (2020), on mind wandering, attentional performance, and well-being. This MBI is called Mindfulness-based Attention Training (MBAT). MBAT was originally developed for US soldiers and research showed that it can be taught to new intervention facilitators via a train-the-trainer programme (Jha et al., 2020). The benefits of being able to train mindfulness facilitators through such a programme is that it may help to train people who are familiar with the military context and are available on site (Jha et al., 2020). The MBAT programme demonstrated beneficial effects on cognitive performance in military settings (Jha et al., 2020; Zanesco et al., 2019). Moreover, recent research that meta-analysed the effects of studies using MBAT shows positive effects on working memory performance (Jha et al., 2022).

In our present study, we aim to investigate the application and effects of MBAT in the Royal New Zealand Navy (RNZN). The RNZN is a service within the New Zealand Defence Force (NZDF). Like other modern militaries, NZDF must be prepared to engage in a variety of complex and dissimilar roles along the conflict spectrum; from civil defence and domestic support duties, humanitarian and disaster relief, to peacekeeping, counterinsurgency, to full scale warfighting at the combat end of the spectrum (Kaspersen, 2021; New Zealand Defence Force, 2017). The Aotearoa/New Zealand geostrategic environment is noted to be deteriorating, with greater strategic competition in the Indo-Pacific and climate change impacting the Pacific likely to require greater NZDF involvement, and therefore pressure on NZDF personnel, in the future (New Zealand Ministry of Defence, 2021). In this feasibility study we evaluate the potential of MBAT to protect attentional performance, associated mind wandering and well-being in a novel setting with newly recruited naval military personnel in New Zealand. In summary it is hypothesised that MBAT could protect from performance decline over time and enhance well-being in demanding training conditions.

## Method

### Participants

Participants were 24 newly recruited Junior Officers (JOs) of the RNZN who were undergoing the Junior Officer Common Training (JOCT), which is a demanding 6-month training programme to prepare them for military service in the RNZN. The JOs were a convenience sample who were approached to participate in this study on a voluntary basis. The mean age of the sample was 24 years (SD = 8.47) and 63% were male. Only participants who completed measures at all assessment points were included in the analysis, resulting in a final sample of *n* = 17.

## Ethics

Ethical approval for this research was given by the authors’ institutional ethics committees. While MBAT was incorporated as part of the JOCT programme, participation in the research element was voluntary, entirely anonymous and participants could withdraw at any point. In order to be able to match data across time points while ensuring anonymity, participants were asked to create a unique code consisting of their parents’ initials and their day of birth.

### Procedure

**Figure 1.**
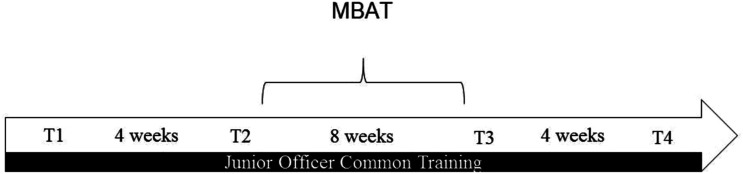
Study design.

The JOCT is a 6-month long course and MBAT was delivered during months three and four. A measure of well-being as well as the SART were administered at four time points: 4 weeks prior to the start of the MBAT (T1), immediately prior to the MBAT (T2), upon completion of the 8-week MBAT (T3), and a follow-up assessment 4 weeks after completion of the MBAT (T4). Well-being measures were administered using a paper and pencil format and the SART was conducted via laptops using the open source software package PsychoPy. An illustration of the study design can be seen in Figure 1.

The MBAT protocol is a manualized program initially developed for military populations (Denkova et al., 2020; Jha et al., 2020; Zanesco et al., 2019). This protocol contains four central themes that have corresponding mindfulness exercises. The first theme to be introduced is *concentration*, which includes a guided concentration meditation asking participants to focus on their breathing, and return their attention back to their breathing when mind wandering is observed. The second theme is *body awareness*, which is trained through a guided body scan, instructing participants to notice any sensations in the body while being non-judgmental of any sensations that arise. The third theme is *open monitoring*, which aims to enhance awareness and receptiveness to changing circumstances and asks participants to expand their level of awareness without a designated object of attention. The last theme is *connection*, which targets relationship and group cohesion by asking participants to engage in kindness and compassion towards oneself and others (Zanesco et al., 2019). The MBAT protocol can be delivered as 4 2-h sessions, or as in this study as 8 1-hr sessions. MBAT differs from other standardised MBI protocols, such as MBSR (Kabat-Zinn, 1990) which delivers 2.5 hours of contact sessions over a period of 8 weeks and is therefore considered a low-dose intervention. This programme was delivered over a period of 8 weeks with one weekly 1-hour class. In addition to that, participants were encouraged to practice meditation 15–20 minutes daily as part of homework. The developers of MBAT trained staff at the New Zealand Defence Force, who in turn trained a staff member from the RNZN to deliver the programme through a train-the-trainer initiative. Jha et al. (2020) provide a detailed outline of MBAT and the train-the-trainer procedure.

### Measures

#### Sustained attention

This study used a modified version of the SART as used by Zanesco et al. (2019). Participants were placed in front of laptops and were presented numbers (0–9) for 250ms followed by an inter-trial period presenting a fixation cross for 900ms. They were instructed to press the space bar when any number except number three was presented and to refrain from pressing the space bar when seeing number 3. The SART consisted of 27 target (number 3) trials, 519 non-target trials and 28 probe questions. The probe questions consisted of two consecutive, randomly distributed questions, forming 14 pairs and served the purpose of capturing spontaneous moments of mind wandering. Probe one asked “Where was your attention focused?” which could be answered on a 6-point Likert scale using the keyboard (1 = on task, 6 = off task). Probe two asked “How aware were you of where your attention was?” which could also be answered using a 6-point Likert scale (1 = aware, 6 = unaware). Participants completed a 163-trial practice block first to familiarise themselves with the task. This practice block was not included in the analysis section. In order to assess performance on the SART three outcomes were investigated, namely *accuracy*, *reaction time variability*, and *subjective mind wandering*.

The index for *accuracy*, known as *A′*, is regarded as a valid indicator of objective task performance (Jha et al., 2015; Zanesco et al., 2019). *A′* was calculated entering the number of hits (correctly withholding from pressing space bar for targets), misses (incorrectly pressing the space bar for targets), false alarms (incorrectly withholding from pressing the space bar for non-targets) and correct rejections (correctly pressing space bar for non-targets) of each participant into the Signal Detection Calculator (v.1.1.1.) provided by Gaetano et al. (2015). The resulting index *A′* was then entered in SPSS for each participant. *Reaction time variability* for each participant was indexed through the intra-individual coefficient of variation (ICV), which is the quotient of the standard deviation of the reaction time of correct non-targets and the mean reaction time of correct non-targets (Zanesco et al., 2019)

$$\frac{SD\;reaction\;time\;non-targets}{M\;reaction\;time\;non-targets}$$

A higher ICV indicates that response times vary to a greater extent. Previous research states that the ICV is a valid objective indicator of mind wandering (Seli et al., 2013). Finally, *subjective indicators of mind wandering* were measured using mean scores for each of the two probe questions.

#### Well-being

Well-being was measured using the short Warwick Edinburgh Mental Well-being Scale (SWEMWBS; Stewart-Brown et al., 2009). The full version of the scale was found to be valid (Tennant et al., 2007), and the shortened version applied the Rasch model to enhance precision and reliability of the scale showing strict unidimensionality (Stewart-Brown et al., 2009). The measure includes seven items and can be rated on a 5-point Likert scale (1 = none of the time, 5 = all of the time). Reliability was good in the present study (α T1 = .75, α T2 = .89, α T3 = .86, α T4 = .90). Items were summed with a higher score indicating higher levels of well-being. All raw scores of the SWEMWBS were transformed into metric scores for data analysis using Rasch score transformation tables as suggested by the authors (Stewart-Brown et al., 2009).

### Data Analyses

Data analysis was conducted using IBM SPSS v26. Descriptive statistics and Pearson correlation coefficients were computed to investigate relationships. Changes in variables under investigation were analysed over time controlling for baseline (T1) using a repeated measures ANOVA. Changes in variables were created by subtracting baseline values from variable values at each time point.

## Results

Descriptive statistics of the outcome measures as well as correlations can be found in Tables 1 and 2. Subjective as well as objective reports of mind wandering assessed via the ICV (T1 *r* = −.70, *p* < .01) and probe questions 1 (T1 *r* = −.31, *p* = .23) and 2 (T1 *r* = −.38, *p* = .15) had moderate to strong negative associations with accuracy on the SART, which is in line with the theory.

**Table 1.** Descriptive statistics with mean and standard deviation for the outcome measures across four time points.

	T1	T2	T3	T4
A′	.86 (.15)	.91 (.12)	.90 (.14)	.84 (.19)
ICV	.59 (.28)	.46 (.22)	.50 (.24)	.53 (.21)
MW probe 1	2.65 (1.35)	2.51 (1.29)	2.45 (1.00)	2.79 (1.43)
MW probe 2	2.27 (0.93)	2.43 (1.25)	2.39 (1.36)	2.69 (1.41)
Well-being	22.57 (4.39)	24.85 (4.99)	24.07 (3.92)	26.51 (4.45)

*Note.* Calculation of A′ and the ICV explained in the method section; MW = Mind wandering, probe one and two reflect mean scores respectively; Well-being (SWEMWBS).

**Table 2.** Correlations between outcome measures at T1.

	A′	ICV	Probe 1	Probe 2
ICV	−.70^a^			
MW probe 1	−.31	.21		
MW probe 2	−.38	.22	.66^a^	
Well-being	.15	.12	.25	.14

^a^*p* < .01. *Note*. Calculation of A′ and the ICV explained in the method section; MW = Mind wandering, probe one and two reflect mean scores respectively; Well-being (SWEMWBS).

**Figure 2.**
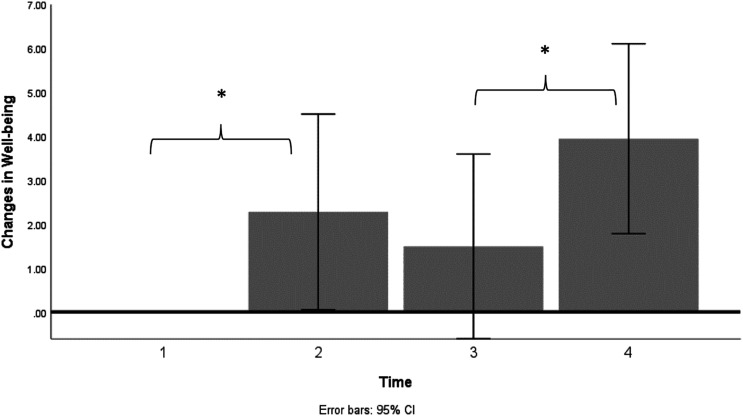
Changes of well-being over time with the reference to the baseline set up to zero. Note. *p<.05.

Figure 2 illustrates changes in well-being over time with reference to baseline. It can be seen that increases in well-being are evident at all three time points after baseline, showing continuous improvement in well-being levels. A repeated measures ANOVA indicates that those changes in well-being over time were statistically significant (*F* (3,48) = 6.72, *p* = .01) with a large effect size ( $\eta_{p}^{2}$ = .30). Pairwise comparisons between time points were made using paired samples t-Tests, which showed that changes in well-being were significant from baseline to T2 *t* (16) = –2.17, *p* < .05, *d* = 0.52 and from T3 to T4 *t* (16) = –4.25, *p* < .01, *d* = 1.03, both with a large effect size. The significant well-being changes after the intervention with a large effect size strongly supported our hypotheses. No significant performance decline was observed for accuracy while objective and subjective indicators of mind wandering as assessed through the ICV and the two probe questions remained stable across all time points as evidenced by repeated measures ANOVA (all *p-*values >.05). A stable level of these attentional performance and mind wandering indicators is in line with predictions and indicated that MBAT might have protected from performance decline.

## Discussion

The aim of the current feasibility study was to analyse the impact of an established low-dose MBI called MBAT on attentional performance, mind wandering and well-being, delivered by a facilitator who was taught the programme through a train-the-trainer initiative. Results indicate that MBAT is feasible in the New Zealand military context with positive effects on well-being and possible protective effects on performance.

Demanding training, such as that provided in military settings, can have a negative impact on attentional performance, and MBAT may protect from performance decline (Jha et al., 2015). Our findings also suggest that MBAT might help to protect from attentional performance decline for participants undergoing demanding training. Performance in terms of accuracy on the SART remained constant with no significant change at the start of the MBAT until the end of MBAT and at the 4-week follow-up. No significant changes for subjective and objective indicators of mind wandering through the probe questions and the ICV were evident. The non-significant results of subjective mind wandering are not surprising considering that individuals are often not aware of their own mind wandering due to a lack of meta-awareness (Schooler et al., 2011) and can probably therefore not accurately report it.

Well-being consistently improved over time: even though no immediate improvement was evident upon completion of the intervention, well-being significantly increased at the 4-week follow up. Such delayed well-being effects are also documented in the literature. For instance, it was found that an MBI with children had a greater effect reported at a follow-up assessment compared to well-being effects immediately upon completion of the intervention (van de Weijer-Bergsma & Langenberg, 2014). A possible explanation for this delayed effect was suggested in a study which noted that students with higher levels of mindfulness at the start of a semester reported higher well-being at the time of the final semester test due to the tendency to appraise future events in a non-threatening manner (Weinstein et al., 2009). This adaptive way of coping with stress may need some time before it is manifested in the individual’s life and this could explain why well-being effects are higher at the 4-week follow-up (van de Weijer-Bergsma & Langenberg, 2014). It is also possible that well-being improved at T4 follow-up, due to further encouragement to practice mindfulness through apps and groups.

Well-being also significantly improved from T1 to T2, which was prior to the start of MBAT. A possible explanation of these findings is that the RNZN JOs had just started their military career and training a few weeks prior the first data collection point. In the weeks before our T1 measure JOs were already exposed to multiple stressors and intensive training (such as learning of general service knowledge, uniform maintenance, long working hours, physical training sessions and drills, and a week-long camp that aimed to fatigue them). This could well have negatively affected their T1 well-being and the observed improvements over the 4 weeks to T2 could reflect natural adjustment to military life. In addition, physical demands decreased and cognitive demands increased during this time, which could have been more in the comfort zone of JOs. And finally, there was a corresponding reduction in workload due to the onset of the COVID-19 pandemic at that point, which could also have positively affected T2 well-being.

The findings of the present study have implications for new military recruits in training. Considering that many new military recruits often experience high levels of stress (Cigrang et al., 2000), which are likely to impact their performance and well-being, it is imperative to offer interventions that may help to protect against performance and well-being deterioration. The present research shows that a low-dose MBI can help to sustain attentional performance at a high level and even shows a positive impact on well-being at follow-up. MBAT may therefore be an element that could be considered part of the training schedule for new military recruits. Moreover, this feasibility study provides evidence that military personnel can be trained to deliver an established MBI.

These findings are important from a contextual perspective. New military personnel enter an environment that is largely different from what they have known, and they are disconnected from their immediate social network. In addition to that, they must navigate life in a highly challenging work environment where they are required to acquire new skills quickly. These factors may contribute to increased levels of stress and lower levels of well-being, and it is important to provide mechanisms that enable them to cope with these demands, which could possibly also help to reduce the number of individuals who quit military training.

This research has a few limitations that need to be considered. The improvements in well-being in the pre-intervention period and at follow-up are difficult to interpret and any possible explanations are speculative. Future studies with a similar design should include control measures to capture any other influencing factors that could have had an impact on these variables, such as how study participants were adjusting to military life or their levels of fatigue. Controlling for such variables will help to interpret changes that cannot be attributed to the intervention. The inclusion of a control group which either receives no MBAT or an alternative intervention would be useful to draw causal conclusions regarding the effects of the intervention. For instance, the delayed effect on well-being could be better interpreted in comparison to a control group of the same military cohort. If well-being levels increased for both groups, it would be highly likely that the well-being increase cannot be attributed to the intervention but other factors in their military training environment. Moreover, the attrition rate presented a challenge, and the onset of the pandemic might have been a contributing factor to that.

Moreover, this study did not control for mindfulness home practice. Participants were given a practice log where they were asked to record practice minutes outside formal contact sessions, however, the return rate was so low that this could not be included in the analysis. Encouraging participants to record their practice time and to return it at the end will help to control for the influence of informal home practice.

In summary, this study showed that MBAT has the potential to protect new RNZN JOs from attentional performance deterioration while attending demanding military training. The MBAT also had a positive effect on well-being 1 month after completion. It could therefore be considered for inclusion in military training and may be beneficial for performance and well-being outcomes.
